# #Vape: Measuring E-Cigarette Influence on Instagram With Deep Learning and Text Analysis

**DOI:** 10.3389/fcomm.2019.00075

**Published:** 2020-01-22

**Authors:** Julia Vassey, Catherine Metayer, Chris J. Kennedy, Todd P. Whitehead

**Affiliations:** The UC Berkeley Center for Integrative Research on Childhood Leukemia and the Environment, Berkeley, CA, United States

**Keywords:** vape, vaping, e-cigarettes, social media, instagram, deep learning, images

## Abstract

E-cigarette use is increasing dramatically among adolescents as social media marketing portrays “vaping” products as healthier alternatives to conventional cigarettes. In September 2018, the Food and Drug Administration (FDA) launched an anti-vaping campaign, in U.S. high schools, on social media and other platforms, emphasizing “The Real Cost” of e-cigarettes. Using a novel deep learning approach, we assessed changes in vaping-related content on Instagram from 2017 to 2019 and drew an inference about the initial impact of the FDA’s Real Cost campaign on Instagram. We collected 245,894 Instagram posts that used vaping-related hashtags (e.g., #vape, #ejuice) in four samples from 2017 to 2019. We compared the “like” count from these posts before and after the FDA campaign. We used deep learning image classification to analyze 49,655 Instagram image posts, separating images of men, women, and vaping devices. We also conducted text analysis and topic modeling to detect the common words and themes in the posted captions. Since September 2018, the FDA-sponsored hashtag #TheRealCost has been used about 50 times per month on Instagram, whereas vaping-related hashtags we tracked were used up to 10,000 times more often. Comparing the pre-intervention (2017, 2018) and post-intervention (2019) samples of vaping-related Instagram posts, we found a three-fold increase in the median “like” count (10 vs. 28) and a 6-fold increase in the proportion of posts with more than 100 likes (2 vs. 15%). Over 70% of Instagram vaping images featured e-juices and devices, with a growing number of images depicting a “pod,” the type of discrete vaping device that delivers high concentration of nicotine and is favored by novice e-cigarette users. In addition, the Instagram analytics data shared by the vaping influencers we interviewed showed underage Instagram users among their followers.

## INTRODUCTION

Electronic nicotine delivery systems (ENDS), also known as e-cigarettes (National Institute on Drug Use, 2018), are now the most-commonly-used tobacco products among American teenagers (Centers for Disease Control and Preventions, 2019). According to the National Youth Tobacco Survey, the fraction of high-school students who reported using e-cigarettes jumped from 12% in 2017 to 28% in 2019 (Wang et al., 2019). In combination with stagnating declines in the use of other tobacco products, this considerable increase in e-cigarette consumption has reversed recent progress in controlling adolescent tobacco use (The U.S. Food Drug Administration, 2018a; Gentzke et al., 2019; Wang et al., 2019). While the long-term health effects of e-cigarette consumption are not fully understood (American Cancer Society, 2017), e-cigarettes can harm the adolescent brain and increase susceptibility to tobacco addiction (The U.S. Department of Health Human Services, 2016; Fraga, 2019; Wang et al., 2019). As a result, youth who use e-cigarettes are more likely to subsequently try combustible cigarettes (The U.S. Food Drug Administration, 2018a). Beyond addiction, e-cigarettes pose a risk of breathing difficulties, inflammatory reactions, lowered defense against pathogens and lung diseases (Centers for Disease Control and Prevention, 2019a,b; The U.S. Food and Drug Administration, 2019d).

The design and portability of e-cigarette devices contribute to their growing popularity among young people. E-cigarettes deliver high concentrations of nicotine from a device the size of a USB flash drive (Supplemental Figure 1) while producing a *discreet* vapor cloud. Pod devices with disposable or refillable cartridge-based systems are particularly popular among beginner users due to their simple design and convenience (MistHub, 2015; Matt, 2018; Fraga, 2019). More experienced users may opt for larger tank systems (also called mods, box mods or subohm devices) (MistHub, 2015; Smoketastic, 2016) that must be manually-refilled with liquid (also called E-juice or E-liquid).

Young adults are exposed to e-cigarette endorsements from peers and influencers (Carbone, 2018; Dyer, 2019; The U.S. Food and Drug Administration, 2019b; VaporDNA, 2019), on social media. Exposure to social media marketing and other forms of visual advertising have been associated with increased e-cigarette use among adolescents (King et al., 2016; Maloney and Cappella, 2016; Pokhrel et al., 2017; Kim et al., 2019b; Wang et al., 2019). Instagram is one platform popular with U.S. teenagers and young adults (Lee et al., 2015; Smith and Anderson, 2018; Clement, 2019) through which e-cigarette brands have used visual advertising to market their products (Philips, 2018; Hatchinson, 2019). Tobacco companies like JUUL (which controls 70% of the U.S. e-cigarette market) (Centers for Disease Control and Prevention, 2018; Richtel and Kaplan, 2018; Huang et al., 2019; Sherman, 2019) have used Instagram to promote vaping and to brand their products as safe alternatives to conventional cigarettes (Brodwin, 2018; Dyer, 2019), perpetuating a “cost-free” mentality (The U.S. Food Drug Administration, 2018a) toward e-cigarettes. In one national survey of high-school students, nearly 80% of the respondents perceived “no great risk of harm from regular use of e-cigarettes” (Johnston et al., 2019). One common misconception among users is that e-cigarette aerosol consists of innocuous water vapor (Richter, 2018). In reality, e-cigarette vapor can contain several harmful substances, including nicotine, lead, volatile organic compounds, and cancer-causing agents^1^.

In September 2018 the U.S. Food and Drug Administration (FDA) launched “The Real Cost” Youth E-Cigarette Prevention Campaign in U.S. high schools as well as on social media platforms, including YouTube, Spotify, Pandora, Facebook and Instagram. The campaign aims to educate nearly 10.7 million youth aged 12–17 about the hazards of e-cigarettes. It is a nearly $60 million effort funded by fees collected from the tobacco industry (The U.S. Food Drug Administration, 2018a).

To further discourage youth tobacco use (The U.S. Food Drug Administration, 2018b), in November 2018, the FDA announced a restriction on fruity- or sweet-flavored e-liquids sales, allowing them to be purchased only at age-restricted stores or through online merchants that use age-verification (Kaplan and Hoffman, 2018; Kirkham, 2018; Morean et al., 2018; Kaplan, 2019), and a requirement that all ENDS must “bear the addictiveness warning statements on product packages and advertisements” including on social media (The U.S. Food and Drug Administration, 2019e). Despite this regulation, social media influencers have continued to post vaping content on behalf of e-cigarette manufacturers, often failing to include the required nicotine warning (The U.S. Food and Drug Administration, 2019b).

Here we provide a descriptive analysis of Instagram content related to e-cigarettes and comment on the initial impact of the FDA’s social media intervention to reduce vaping among youth. We analyze samples of Instagram posts BEFORE and AFTER the FDA introduced The Real Cost campaign and other anti-vaping measures to evaluate changes in the volume, themes, and user-engagement (number of likes) of vaping-related posts.

## MATERIALS AND METHODS

This mixed method study consisted of two parts: (1) Deep learning analysis of images and captions from Instagram posts; (2) focus groups with young social media users and interviews with Instagram vaping influencers. The goal of the focus groups and interviews was to reinforce, validate, and contextualize the data analysis. The study was approved by the Internal Review Board of the University of California, Berkeley Committee for Protection of Human Subjects (CPHS).

### Qualitative and Quantitative Analysis of Instagram Posts

#### Instagram Data Collection

We collected vaping-related Instagram posts before and after the FDA started its anti-e-cigarette campaign and compared the pre-intervention (2017, 2018) and post-intervention (2019) samples. The posts were obtained by accessing the Instagram Application Programming Interface (API) using subscriptions to web-based applications designed for hashtag tracking. Access to the 2019 data was provided by the social network analyzer Keyhole (Toronto, Canada). The 2017 and 2018 data were collected as part of a prior study (Laestadius et al., 2019) that used the social network analyzer Netlytic (Toronto, Canada) and provided to the authors by the University of Wisconsin-Milwaukee.

**FIGURE 1 ∣:**
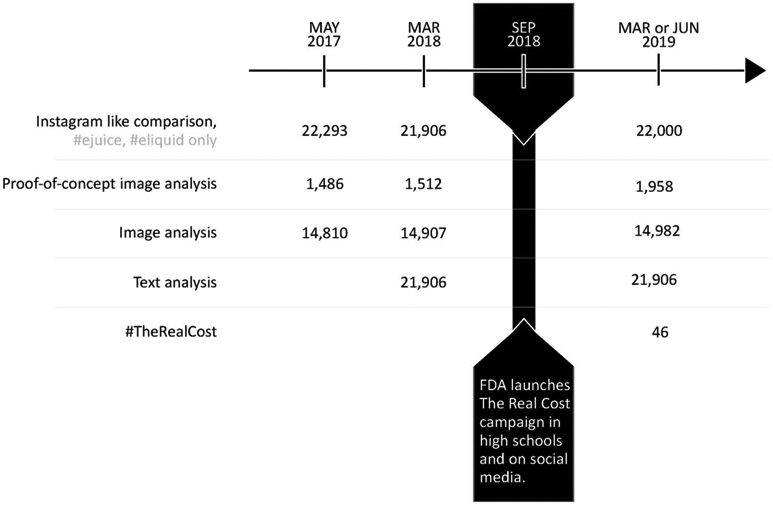
Timeline for the collection of vaping-related Instagram posts.

For the 2019 sample, we collected **201,703** publicly available posts by tracking the following Instagram hashtags which were linked to the promotion and endorsement of vaping in previous content analysis (Laestadius et al., 2016, 2019; Chu et al., 2017): #vape, #ejuice, #eliquid, #vapecommunity, #vapefam, #vapelife, #vapelyfe, #vapenation, #vapeporn. We also collected **46** posts from the FDA campaign by tracking the hashtag #TheRealCost (Figure 1).

For the 2017 and 2018 samples (**22,293** and **21,906** publicly available posts, respectively), Instagram posts were collected only if one of two hashtags—#ejuice or #eliquid—was among the terms used in the caption; however, at least several other hashtags (e.g., #vape or #vapenation) were always present as well.

Instagram posts were collected in real time during sampling periods of <2 weeks. Posts were captured 24 h a day at a rate determined by the API limit of about 100 posts per hour. The collected data included the URLs for images and videos, the date and time of posting, the like count and captions for each post.

#### Number of “Likes” on Instagram

“Likes” is a metric of user engagement (Chu et al., 2017; Boogaard, 2018; Sunshy Group of Companies, 2018) and an important indicator of a post’s impact (Sherman et al., 2017; Martínez-Pecino and García-Gavilán, 2019). We compared the median *“like”* count and the proportion of posts with more than 100 “likes” on Instagram posts before and after the FDA campaign. For the purposes of this comparison, we replicated the protocol which was previously used by Laestadius et al. to collect the 2017 and 2018 Instagram samples (Laestadius et al., 2019), by restricting our June 2019 sample to the 22,000 Instagram posts containing #ejuice or #eliquid as one of the terms in the post’s caption (Figure 1).

#### Image Analysis

We performed deep learning image classification analysis of #ejuice and #eliquid posts from the 2017 sample (*N* = 14,810), the 2018 sample (*N* = 14,907) and the June 2019 sample (*N* = 14,982). These samples excluded any images that were removed from Instagram by the posts’ authors during the data collection period (and prior to analysis). Based on image classification themes identified in the previous studies of vaping posts on Instagram (Laestadius et al., 2016, 2019; Chu et al., 2017) and the description of vaping devices available in the literature (MistHub, 2015; Smoketastic, 2016; Goniewicz et al., 2019), images were divided into the following six categories: (1) ***man*** and (2) ***woman*** (facial images of men or women, usually posing with vaping devices or vape juice bottles or exhaling smoke clouds), (3) ***mod*** and (4) ***pod*** devices, (5) ***e-juice***, and (6) ***other***. Male or female hands holding a vaping device or a bottle of e-juice were assigned to the appropriate vaping device categories.

Deep learning image classification analysis was based on the convolutional neural networks (CNN) framework (The University of Stanford, 2013; Keras, 2015; Deshpande, 2016; Zhang et al., 2018) that recognizes and classifies images by extracting features from an input image (as a matrix of pixel values) and generates a probability distribution over possible output classes (e.g., 0.80 for a man, 0.15 for a mod system device, and 0.05 for e-juice). For each image we selected the output class that had the highest predicted probability in our image classification analysis. We applied transfer learning and fine-tuning (TensorFlow, 2017) to create a customized image classifier capable of recognizing and classifying vaping images. We used the existing CNN called Inception v3^2^ trained by Google on an image dataset consisting of over 1,000,000 images of about 1,000 different classes (ImageNet Library, 2016) as the basis for building our own classification model. We fine-tuned Inception v3 on a set of images identified on Google Images and Instagram using the query terms corresponding to the six identified classes (i.e., *“man” “woman” “mod” “pod” “e-juice” and “other”)* and obtained via a Google Chrome extension downloader (Chrome Web Store, 2019), which allows for the rapid download of large batches of enumerated images. These images were manually reviewed, grouped and labeled according to the six identified classes. The resulting image dataset was divided into a training (1,745 images) and validation set (356 images) at a ratio of 5:1. To fine-tune the neural network we added a 128-unit dense layer, followed by dropout with 60% probability, and then a 6-unit output layer with softmax activation. The model was optimized for categorical cross-entropy loss over 8 epochs using stochastic gradient descent with a learning rate of 0.00001. The model achieved 0.90 validation accuracy and 0.32 validation loss (Supplementary Figure 2). The model was not trained to identify more than one class in a single image. For the images featuring more than one class (e.g., mod, e-juice, group of people), we considered the prediction correct if the classification was accurate for either of the classes.

The fine-tuned model was first evaluated on the smaller set of 4,956 images from the 2017 sample (*N* = 1,486 posts), the 2018 sample (*N* = 1,512 posts), and the March 2019 sample (*N* = 1,958 posts), which were labeled manually. The model correctly classified above 90% of the images with men, women, e-juices, mod system devices across all samples. The model correctly classified 90% of the images with pod system devices in the 2019 sample, 64% of those images in the 2018 sample and 30% in the 2017 sample (Supplementary Figure 3). As a final analysis, we applied the fine-tuned model to generate label predictions on our complete data set: 44,699 unlabeled images from the 2017 sample (*N* = 14,810), the 2018 sample (*N* = 14,907) and the June 2019 sample (*N* = 14,982, Supplementary Figure 4).

We also manually reviewed 1,958 posts for the presence of a warning label and a geographic location, and 4,956 posts for the presence of sexually-explicit imagery.

#### Text Analysis

We applied text mining and natural language processing methods using Tidytext software package in R to analyze and quantify the content of Instagram captions (Silge and Robinson, 2017). We counted the most frequently used words in Instagram captions from the pre- and post-FDA intervention samples. We ignored #eliquid and #ejuice as they were used in every caption (by design) as well as “stop words” such as connecting verbs, prepositions and articles in English and other languages (e.g., “the” “is,” “to”) that are not useful for analysis (Manning and Schutze, 1999). We also analyzed the context surrounding words commonly used in Instagram captions of the #eliquid and #ejuice posts compared to captions of #TheRealCost posts from the March 2019 sample. We used latent Dirichlet allocation (LDA) analysis topic modeling based on unsupervised clustering (Silge and Robinson, 2017). The LDA grouped the dataset of Instagram captions from our sampled posts into a mixture of topics by connecting similar words together and calculating the probability (Beta coefficient) that the grouped words would be part of a selected topic. The desired number of topics and the number of words per topic are user-defined parameters in LDA. Since Instagram captions are sparse and short texts with diverse themes (Hong and Davison, 2010), good results have been achieved in previous studies (Steinskog et al., 2017; Maier et al., 2018) by using a higher number of topics than what is needed for larger, more coherent corpora. We used 50 clusters (K = 50) and a threshold probability for topic inclusion of 2% (Beta = 1/K). We reviewed the words with the highest probabilities for each topic (top words) and attempted to label the substantive content of the topic, as has been done previously (Hong and Davison, 2010; Steinskog et al., 2017; Maier et al., 2018).

### Focus Groups and Interviews With Instagram Influencers

The focus group participants were recruited in March-April, 2019 among undergraduate students of the University of California, Berkeley, and Berkeley City College and among other Berkeley residents who were not students. We included a racially-diverse group of male (*N* = 4) and female (*N* = 4) participants between 18 and 25 years of age who reported that they used social media daily. Although e-cigarette consumption was not an inclusion criterion, four of eight focus group participants reported vaping. The participants were asked to identify their preferred social media platform and the frequency of its use, as well as whether they often see social media posts related to vaping, including the FDA anti-vaping campaign images and videos. The participants were asked to share their impressions of a selection of images and videos related to vaping and the FDA campaign and were asked to describe what content they found most engaging.

We contacted ~100 influencers with public Instagram accounts to request an interview. The list of potential interviewees was seeded with popular influencers referenced in the literature and then expanded via algorithmic Instagram suggestions. Five influencers agreed to be interviewed. The group was comprised of three men and two women; one participant was from the U.K. and the other four were from the U.S. Group member followings ranged from 2,000 to over 200,000 Instagram users (Mean = 73000, *SD* = 74000). During the semi-structured interviews (Robert Wood Johnson Foundation, 2008) the participants were asked about their vaping habits, the motivation to promote vaping products, knowledge of influencers’ marketing strategies and collaboration with vaping brands. They were also asked to share Instagram analytics data, which provided information on the age distribution of their followers.

## RESULTS

As expected, our manual review of the posts and text analysis of the corresponding captions indicated that nearly all of the posts we collected by tracking #ejuice and #eliquid promoted or endorsed vaping. In contrast, the FDA posts featuring #TheRealCost hashtag were the only posts which we identified in our sample that contained anti-vaping sentiments.

### Number of “Likes” on Instagram

**FIGURE 2 ∣:**
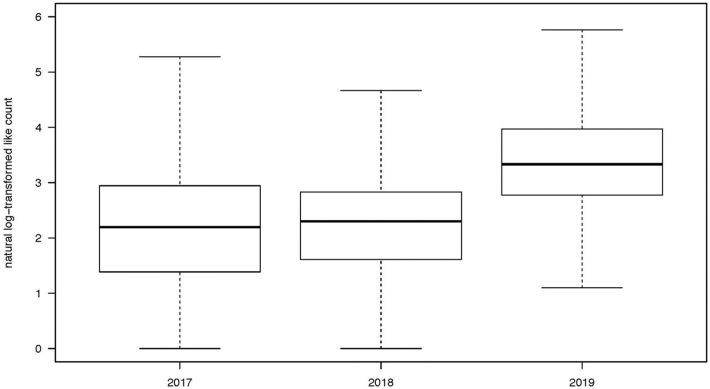
Median like count for vaping-related Instagram posts collected during the 2017 sampling period (*N* = 22,293), during the 2018 sampling period (*N* = 21,906), and the June 2019 sampling period (*N* = 22,000). The lower whiskers are shown as the 25th percentile—1.5*IQR; the upper whiskers are shown as the 75th percentile + 1.5*IQR.

Since August 2018, the FDA-sponsored “anti-vaping” hashtag #TheRealCost has been used about 50 times per month on Instagram, whereas “pro-vaping” hashtags like #ejuice or #eliquid were used 1,000 times more often. Comparing the pre-intervention (2017, 2018) and post-intervention (2019) #e-juice and #e-liquid samples, we found a three-fold increase in the median like count (10 vs. 28 likes) (Figure 2) and a 6-fold increase in the proportion of posts with more than 100 likes (2 vs. 15%). The median like count for the FDA campaign posts was comparable (23 likes) although, again, the volume of posts was much smaller.

### Image Analysis

#### Quantitative Image Analysis

We performed a deep learning image classification analysis of Instagram posts with captions containing hashtags #ejuice or #eliquid from the samples collected in 2017 (*N* = 14,810), 2018 (*N* = 14,907) and June 2019 (*N* = 14,982, Table 1). Over 85% of Instagram vaping images featured *Devices*, and the sub-category *E-juice* was the most prevalent subject in all three samples. The proportion of *E-juice* images remained relatively consistent in 2017 (36%), 2018 (41%), and 2019 (40%). The proportion of *Mod* images decreased in 2019 (21%) compared to 2018 (27%) and 2017 (30%); correspondingly, *Pod* images became more popular, increasing from 2017 (0.9%), to 2018 (2%), to 2019 (9%). We observed a 3-fold increase in the median like count in the 2019 sample compared to the 2017 and 2018 samples for each class of images, which was consistent with the overall 3-fold increase in the median like count presented in Figure 2. Images of women had the highest median like count in all three samples (Table 1).

#### Qualitative Image Analysis

##### Warning labels

Despite the 2018 FDA requirement to add warning labels to tobacco-product advertising on social media, we found that most of the images with the highest like count analyzed in our June 2019 sample (*N* = 1,958 posts, likes > 100) did not contain warning labels in compliance with the FDA requirements (The U.S. Food and Drug Administration, 2019c). The images featuring e-juice had the highest prevalence of warning labels (11%), followed by images of women and pod devices (5%), mod devices (4%) and men (3%). We found fewer than 1% of images with warning labels in both 2017 and 2018 samples, before the warning requirement was introduced.

##### Sexually-explicit imagery

Overall, we found that – among Instagram posts featuring women - about 1 in 8 posts in the June 2019 and 1 in 4 posts in the 2017 and 2018 samples contained sexually-explicit imagery. The median like count for sexually-explicit imagery in the June 2019 sample (106 likes) were twice as high as the median like count for images with no sexually-explicit features (47 likes). In posts from 2017 and 2018, we found no difference in the median like count for sexually-explicit imagery compared to fully clothed women.

### Text Analysis

Several *E-juice*-related words were among the most frequently used in Instagram captions from the 2018 and March 2019 samples, with words like *“juice,” “strawberry” “flavor,” “liquid,” “sweet” “eliquid”* and *“60 ml”* all used at least 500 times. Other words that were frequently used in both samples included *“mod”* and *“kit.”* The words *“Nicotine” “pod” “salt”* (Innes, 2018) and *“nic”* appeared to grow in popularity from 2018 to March 2019. A comparative analysis of words that were used in the #TheRealCost campaign (46 posts, including the FDA and users’ comments), vs. words that were used in the #e-juice and #e-liquid posts showed very little overlap in the March 2019 sample (21,906 posts). Even in instances where a common lexicon could be identified (e.g., with frequently-used words like *“Nicotine” “time” “brain” “addictive” “crave”* and *“stop”)*, the context was different. In captions from the #TheRealCost posts these words were used to warn of health consequences; whereas in captions from the #e-juice and #e-liquid posts these words promoted vaping content, promising a “high” and fun experience. The word *“nicotine”* was used both as a warning label and as a promotion in the pro-vaping captions (Table 2). The March 2019 sample included twice as many non-English-language topics (13 out of 50) compared to the 2018 sample (6 out of 50), including topics in German, Spanish, Italian, French, Malay, Indonesian, Japanese.

**FIGURE 3 ∣:**
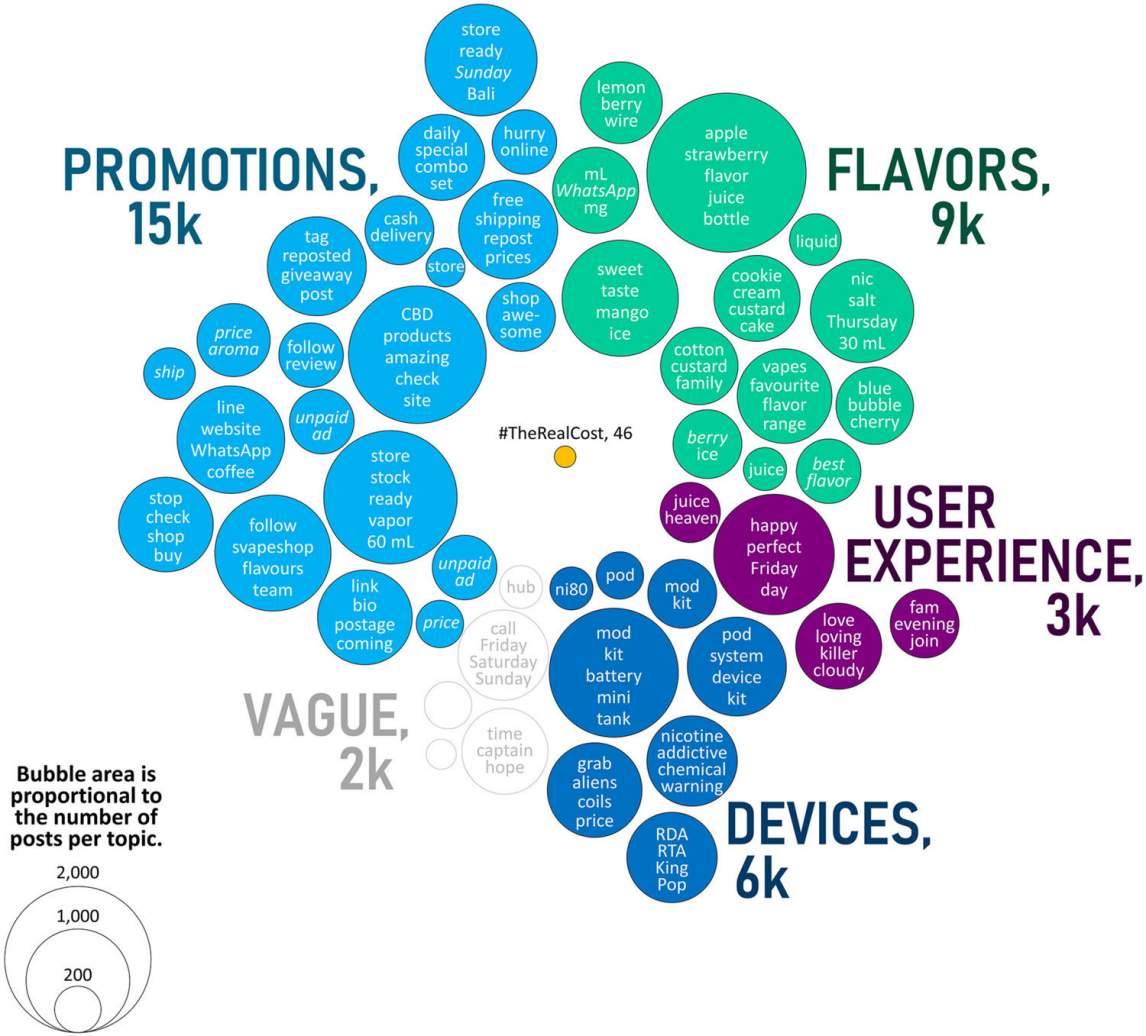
The number of Instagram posts from the March 2019 sampling period belonging to each topic as defined by latent Dirichlet allocation analysis of caption text. Here, topics are further organized into informal themes based on the five most-commonly used words from each topic (representative words are displayed). For comparison, only 46 posts in this sample used the hashtag #TheRealCost. Words shown in italic font were translated to English from the original caption text composed in another language (i.e., from Spanish, German, French, Italian, Malay, or Indonesian).

Based on the most commonly-used words from each of 50 caption topics detected via the latent Dirichlet Allocation analysis (LDA) we identified four themes among the posts of March 2019: (1) ***promotions*;** (2) ***flavors*;** (3) ***devices*;** and (4) ***user experience*** (Figure 3). The themes in the May 2018 sample were very similar, except for the absence of *Pod* system-related and nicotine salts-related topics. LDA detected only pro-vaping topics. No anti-vaping topics were detected by LDA, presumably due to the relatively small number of anti-vaping posts (*N* = 46, Figure 3) identified in the #TheRealCost sample only.

### Focus Groups and Interviews With Instagram Influencers

Some focus group participants described e-cigarette users who follow vaping brands and influencers on Instagram as representatives of a sub-culture, different from mainstream e-cigarette users. They also viewed e-cigarette consumption as distinct from vaping saying that those who smoke e-cigarettes use more consumer-oriented, small, “low key” devices like JUUL, which is “cool” and not as “in your face” as vaping (Table 3). However, Instagram videos featuring smoke-cloud exhalation, aka *vaping tricks*, presented during the focus groups elicited the most positive reaction, while several videos and images related to the FDA intervention were perceived more negatively (Table 3).

Vaping Influencers reinforce the tobacco industry’s sales pitch that e-cigarette use is a healthier alternative to conventional combustion-cigarette smoking. We inferred from their answers (Table 4) that vaping youth are motivated to become influencers and promote vaping products on Instagram by financial incentives. The interviews also suggested an increasing growth of vaping content on Instagram, confirming the findings of our quantitative analysis. In addition, the influencers we interviewed shared their Instagram analytics (Canning, 2018) data revealing that underage users comprised as much as 16% of their followings (Mean = 9%, *SD* = 7%, Range = 15%).

## DISCUSSION

This is the first study to apply large-scale deep learning image classification to social media posts about e-cigarettes. These deep learning methods allow for a fast, automated identification of Instagram images, which offers savings of time and money. This is also the first study focused on e-cigarettes to combine quantitative and qualitative Instagram content analysis with interviews of vaping influencers and focus groups of young social media users.

The analysis from different time periods (2017–2019) allowed us to assess the change of vaping content on social media during a period of time when both Instagram and e-cigarettes use were growing in popularity among youth (Wang et al., 2019). This study design also allowed us to draw an inference about the initial impact of the FDA’s Real Cost campaign on Instagram.

### Assessing the Apparent Impact of the FDA Anti-vaping Campaign on Instagram

#### Low Frequency of Posts

The FDA’s Real Cost campaign was initially launched in 2014 to discourage smoking and the new anti-vaping focus was announced in September 2018 (The U.S. Food and Drug Administration, 2019a). Since then, pro-vaping Instagram hashtags like #vape were used up to 10,000 times more often than the FDA-sponsored hashtag #TheRealCost. Historically, the pro-vaping hashtags we tracked for this study have accumulated as much as 10 million posts, whereas there have been only 3,129 #TheRealCost posts, including all of the anti-vaping and anti-smoking images and videos ever posted from the official FDA campaign account (~16%) as well as from other users (~84%). This stark imbalance in the volume of posts has caused the FDA message to be overwhelmed by direct and sponsored marketing from the vaping brands.

#### Low Impact on User Interaction With Vaping Images and Low Engagement

When comparing the user interaction with the #e-juice and #e-liquid Instagram posts from before and after the initiation of the FDA Real Cost campaign, we found a large *increase* in the median like count and in the proportion of posts with more than 100 likes. Our results indicate that the number of Instagram users exposed to vaping images and videos could be still growing, despite the FDA’s intervention efforts on social media. Despite accumulating a large amount of followers during the four years of its existence (62,000), the most recent (2017–2019) anti-smoking and anti-vaping images and videos posted by the TheRealCost account have had a low engagement rate (Canning, 2018; Plann, 2019) of just ~ 0.8% per post. For comparison, a good engagement rate on Instagram is considered to be between 3 and 6% (Plann, 2019). Infrequent posting and low engagement have the potential to seriously limit the impact of the FDA’s efforts on social media.

#### Persistence of Predatory Themes

There has been criticism of the predatory practice of e-cigarette and e-juice manufacturers to promote flavored nicotine products that appeal to youth on social media (directly or through influencers) (Associated Press, 2019; Kong et al., 2019). In 2017, Laestadius et al. (Laestadius et al., 2019) conducted a qualitative-content analysis of Instagram posts featuring e-liquid to identify common themes, claims and product promotions. The study found that about 60% of the 1,000 images and videos analyzed contained promotions of e-liquids. We found that *e-juice* remained the most prevalent topic in our samples before and after the FDA campaign, and the number of images featuring *pod* devices (commonly used by beginner e-cigarette users) has been growing. Aesthetically pleasing images of male and female models that could alter young users’ perceptions (Harris and Bardey, 2019) were also frequent among the posts featuring vaping products in our samples. Moreover, Instagram analytics data collected from vaping influencers showed considerable proportions of underage (13–17 year-old) followers, indicating that youth will likely continue to be exposed to vaping content marketed on Instagram. This finding is consistent with a recent Kim et al. (Kim et al., 2019a) which demonstrated that over 40 percent of JUUL Twitter account followers are underage (13–17 years old). Exposure to potentially harmful social media content is even more concerning, as previous studies here demonstrated that teenagers often relate to Instagram influencers more than to their physical friends (Mañas-Viniegra et al., 2019).

#### Non-compliance With Warning Label Requirements

Despite the 2018 FDA requirement that all ENDS “bear the addictiveness warning statements on product packages and advertisements” on social media, we found that, overall, only about 7% of the posts in our June 2019 sample contained warnings in compliance with the FDA requirements (The U.S. Food and Drug Administration, 2019c). Posts uploaded from locations within the U.S. had the highest prevalence of warning labels, while posts uploaded from other countries were less likely to include warnings. Most of the international posts featured vaping products distributed in the U.S. and would therefore still be subject to compliance with the FDA warning-label regulations (The U.S. Food and Drug Administration, 2019c). This finding indicates that there should be increased enforcement of the warning-label regulation, especially among the international network of influencers, distributors and retailers.

### Participatory Strategies for Discouraging Youth Vaping

One major challenge facing the FDA’s anti-vaping campaign is the common perception among youth that vaping is a harmless activity (Kim et al., 2019b; Wang et al., 2019), a misconception perpetuated by tobacco-industry marketing (Huang et al., 2019). In an attempt to disabuse teenagers of this “cost-free” mentality toward vaping (The U.S. Food Drug Administration, 2018a)^1^, the FDA campaign features images and videos of distorted faces and brains that are meant to reveal the real (but hard to see) detrimental health effects (The U.S. Department of Health Human Services, 2016, 2017; American Cancer Society, 2017; Chung et al., 2018; Centers for Disease Control and Preventions, 2019; Gentzke et al., 2019; Wang et al., 2019) of e-cigarette consumption. Most of our focus-group participants found these images and videos repulsive and scary; however, seeing them did not motivate any e-cigarette users in our group (*N* = 4 of 8) to quit vaping. Generally, appealing to fear in order to raise awareness about health concerns such as smoking can be a valid approach if the messaging is based on evidence or reason (Simpson, 2017). However, fear-inducing tactics can only be effective when an intervention target is perceived as a threat (Albarracin et al., 2005). While smoking is viewed by many consumers as a risky behavior, vaping is not. Some Instagram users responded to the FDA warnings about the danger of vaping aerosol with comments disputing the FDA claims of damaging health effects from nicotine or by asking public health officials to provide a list of toxic chemicals that are present in e-cigarettes as a proof of their harmfulness. The users also referred to the FDA campaign as “propaganda” that people should not take “seriously” and suggested that the campaign’s anti-vaping ads just scared people without actually encouraging them to quit vaping.

Previous research (Albarracin et al., 2005) indicates that participatory, active intervention strategies could be more effective than passive educational campaigns. Indeed, our focus-group participants suggested that sharing vaping experiences in group discussions might be an alternative cessation strategy. Similar interventions were described as effective elsewhere (Ramo et al., 2014). Vaping brands are already using participatory strategies to effectively engage both legal-age and underage vapers as social-media promoters and brand ambassadors. In exchange for posting images featuring vaping products on social media, brands offer influencers commissions on sales of vaping products (VaporDNA, 2019), promotional giveaways and prospects of online exposure. Our interviews indicate that vaping enthusiasts and influencers view these incentives as alluring. Perhaps, public health officials could use participatory interventions to thwart the vaping brands’ marketing strategies and engage youth in developing and market-testing anti-vaping messages.

### Legislative Intervention for Discouraging Youth Vaping

National, state, and local programs have been shown to reduce and prevent youth tobacco use (Centers for Disease Control and Preventions, 2019), for example by raising taxes for tobacco products or by raising the minimum age of sale to 21 years. The legal minimum age to purchase e-cigarettes differs by state, ranging from 18 to 21, but a majority of states have no legislative restrictions in place prohibiting the *use* of e-cigarettes by youth who have not yet reached the legal minimum age of sale. Businesses violating restrictions on the sale of e-cigarettes to youth can face a range of minor penalties including a simple warning (in 3 states), a fine (in 31 states), or a misdemeanor criminal charge (in 16 states) (Centers for Disease Control and Prevention, 2019c). Only a handful of states have excise tax on e-cigarette sales (Centers for Disease Control and Prevention, 2019c). Youth E-cigarette use could be further discouraged by raising the legal age for e-cigarette purchase to 21 in all states, by introducing stricter penalties for underage sales or use, and by increasing taxes on products.

## LIMITATIONS AND FUTURE RESEARCH

Although the classification accuracy of the deep learning image analysis was sufficient for descriptive content analysis, it was not perfectly accurate. For device classification, the model was only trained to recognize one class per image, whereas some images had two classes (e.g., *“e-juice”* and *“mod”*). Distinguishing pod and mod systems by machine algorithm was also a challenging task as some traditional mod devices are being redesigned to resemble smaller modern pod devices. Additional model training would be required to enable further deep learning image analyses to conduct the following tasks: more precise gender detection of blurred images and recognition of full-body background, identification of faces obscured by smoke, prediction of more than one class in a single image, and integration of post image pixels and post texts to evaluate compliance with the FDA requirements to add warning labels and sponsorship disclosures.

To assess Instagram-user engagement with vaping posts we focused on “like” count as a surrogate for Instagram-user engagement with vaping posts; however a “like” does not necessarily indicate a user’s approval of or support for e-cigarette use. Still, since “likes” are often perceived by adolescent users as determinants of appropriate social behavior (Sherman et al., 2017), the exposure to social media vaping images with many “likes” could be interpreted by youth as a signal of approval of a risky behavior, e.g., vaping.

We did not calculate engagement rates (Canning, 2018; Plann, 2019). Engagement rate per post depends on the follower count of the poster, data which were not available in our samples due to Instagram’s data-access restriction policy. Similarly, data-access restrictions prohibited us from identifying the geographic locations for ~ 80% of the posts. As data-access policies on social media platforms grow increasingly restricted, collaborations between platforms like Instagram and academic institutions will be necessary to conduct ethical, responsible, and useful research about the impacts of social media.

Our interviews and focus groups indicate that the followers of Instagram vaping accounts might represent a subculture of avid enthusiasts, who enjoy watching vape tricks and monitoring the latest models of vaping devices (mostly *mods* that produce vaping clouds). This group of enthusiasts may not be representative of most young e-cigarette users (including the teenagers targeted by the FDA campaign) who tend to prefer consumer-oriented devises like JUUL *pods* (and other non-sub ohm devices) (Matt, 2018;Tolentino, 2018). Further study is warranted to characterize the broader influence of social media marketing on youth e-cigarette use: by measuring the attention that youth pay to vaping advertising, by analyzing the emotional intensity of young people reacting to these stimuli, and by conducting focus groups and surveys among diverse groups of adolescents to gauge their perceptions of this content. Likewise, because our analysis of the initial impact of the FDA campaign was based on a limited number of Instagram comments and responses from a small number of focus group participants from one location of Berkeley, CA, a more comprehensive study of the effect of the campaign in middle and high schools (The U.S. Food Drug Administration, 2018a) is warranted. Finally, further study is warranted to include interviews with a larger number of vaping influencers.

## Supplementary Material

Supplementary Figure2

Supplementary Figure3

Supplementary Figure1

Supplementary Figure4

## Figures and Tables

**TABLE 1 ∣:** Predicted deep learning image classification for Instagram posts using #ejuice or #eliquid collected in 2017 (*N* = 14,810), 2018 (*N* = 14,907), and 2019 (*N* = 14,982).

	Number (%) of posts in each image/video classification			Number (%) of high-engagement posts (i.e., with 100+ “likes”)			Median “like” count		
Classification of image/video	2017	2018	2019	2017	2018	2019	2017	2018	2019
**FACIAL IMAGES**									
Men	1,153 (8%)	1,096 (7%)	1,118 (7%)	42 (0.3%)	44 (0.2%)	225 (2%)	12	12	34
Women	825 (5%)	775 (5%)	829 (6%)	37 (0.2%)	49 (0.3%)	282 (2%)	13	15	48
**DEVICES**									
E-juice	5,316 (36%)	6,049 (41%)	6,043 (40%)	98 (0.7%)	90 (0.6%)	809 (5%)	9	9	27
Mods	4,404 (30%)	4,070 (27%)	3,102 (21%)	157 (1%)	131 (0.9%)	526 (4%)	10	11	32
Pods	130 (0.9%)	292 (2%)	1,335 (9%)	7 (0.05%)	13 (0.08%)	158 (1%)	8	10	24

Class “Other” is not included in the table.

**TABLE 2 ∣:** Commonly used words in captions of Instagram posts related to vaping from the March 2019 sample (*N* = 21,906) and the U.S. Federal Food and Drug Administration anti-vaping campaign “The Real Cost” (*N* = 46).

Commonly used words	#TheRealCost posts examples	Vaping posts examples
Nicotine	“Vaping can put nicotine in your brain, which can change the way your brain works, causing you to crave more and more.”	“No nicotine” (in posts advertising e-juice without nicotine); “nicotine level: 30 mg bottle.”
Time	“Don’t be a guinea pig in an experiment … only time will tell what the true effects.”	“Giveaway time”; “Get ready for a great time…”
Addictive	“Nicotine—the highly addictive drug in most vapes—can create powerful cravings that make it hard to think about anything else.”	“Warning: This product is intended to be used with e-liquid that may contain nicotine.Nicotine is an addictive chemical.”
Crave	“Over time, nicotine can change the way your brain works, causing you to crave more nicotine and vape more frequently.”	“Crave salt nic now available;” The post also describes craving e-juice.
Brain	“An epidemic is spreading.It can harm your brain.”	“… this “brain coffee” is perfect!”
Stop	In reaction to one of the #TheRealCost posts, one Instagram user urged the campaign to “stop” the ads because they were making the user “cringe.”	“Stop and stare.” (in reaction to e-juice) “Stop smoking today” *(as opposed to vaping.)*

**TABLE 3 ∣:** Quotes from focus groups with 18 to 25-year-old Berkeley residents (*N* = 8).

1.1 [They are] particular kind of people that vape. Not all vapers are the vapers that engage in the vaping sub-culture on social media. [They wear] certain kind of clothing, speak in a certain way. They are usually men, in their teens or 20s. Vapers have skateboard, [they are] skinny.
1.2 JUUL is a new thing. It is like e-cigarettes, it is different than vaping. I see a lot of them on social media and it is not seen in a same way as vapers. JUUL is so ubiquitous, it is everywhere. It becomes normalized, it is like a cool new thing and everyone does it. When people look at you when you smoke a cigarette, they think tha’s bad for you and it is going to give you cancer. JUUL I guess is supposed to be healthier. JUUL is a different device, which produces less smoke and less vape. Design in general. JUUL is like slick. Not like a giant, really weird-looking devices. The design makes JUUL look low key. That is probably what draws people to it.
1.3 [The quote describes a video featuring a vaping influencer making a bubble out of e-liquid] With a bubble is a cool video, like a magic show.
1.4 I do not think this message is effective. It is super like a fancy way of reminding us that nicotine is addictive. I imagine myself scrolling on my phone, trying to enjoy myself and [the FDA anti-vaping campaign] is trying to tell me what to do.

**TABLE 4 ∣:** Quotes from the interviews with the Instagram vaping influencers (*N* = 5).

2.1 [Among vaping enthusiasts], everybody knows someone whose life was changed by vaping. That’s a point of inspiration. For example, if you talk to my mother or my grandmother, they stopped smoking and switched to vaping and now they are healthier. And they are happy they did it.
2.2 I post videos of me doing vape tricks. They [vaping brands] reach out to me because I have a lot of followers and get a decent/good amount of views. If they want promotions then they message me, we work out a deal, they ship products and I make the post requirements. I’ve worked with companies in America, China, The UK and Malaysia. I wouldn’t call myself a model, I just do vape tricks. It’s more like a hobby you make money out of. But within my area there’s about probably 10 people in my area that I’m friends with that do what I do. Guys and girls.
2.3 [Instagram] is like going to a car show to see what’s new. It’s one of the only ways that the manufacturers could show their new products. And there are new products weekly, if not daily. Manufacturers give them away as a mean of advertising. They send me something new—I show it off.

## Data Availability

The 2019 raw data supporting the conclusions of this manuscript will be made available by the authors, without undue reservation and in compliance with the IRB protocol, to any qualified researcher. The 2017 and 2018 data analyzed in this study was obtained from the University of Wisconsin-Milwaukee. Requests to access these datasets should be directed to Linnea Irina Laestadius, llaestad@uwm.edu.

## Footnotes

1 Centers for Disease Control and Preventions (2019): About Electronic Cigarettes. Last reviewed November 15, 2018. https://www.cdc.gov/tobacco/basic_information/e-cigarettes/about-e-cigarettes.html.

2 Google Cloud: Advanced Guide to Inception v3 on Cloud TPU. https://cloud.google.com/tpu/docs/inception-v3-advanced.
