# Supplementary figures and images for: #Vape: Measuring E-Cigarette Influence on Instagram With Deep Learning and Text Analysis
Source: Front Commun (Lausanne). Author manuscript; Available in PMC 2022 Feb 28. (PMC8883232; doi:10.3389/fcomm.2019.00075)

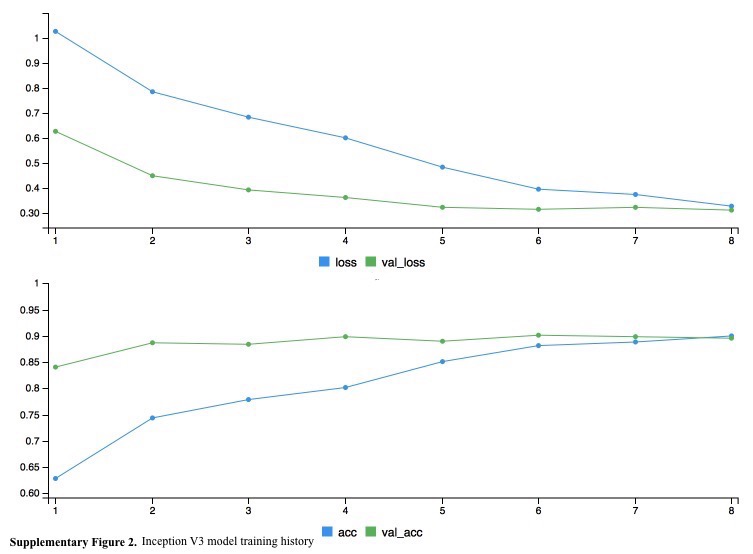

Supplement: Supplementary Figure2 [file NIHMS1776832-supplement-Supplementary_Figure2.jpeg]

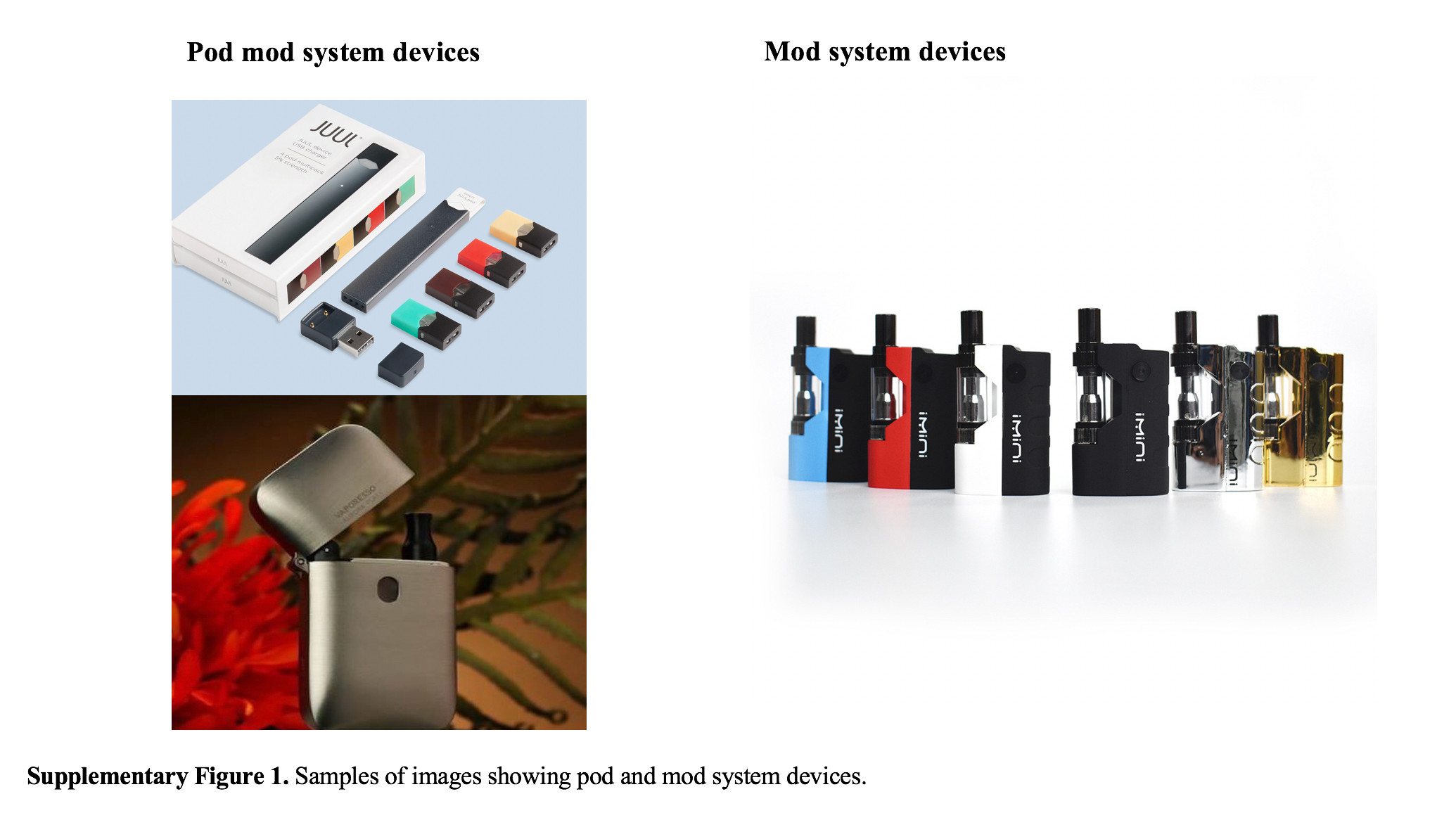

Supplement: Supplementary Figure1 [file NIHMS1776832-supplement-Supplementary_Figure1.jpg]
