# Supplementary material for: #Vape: Measuring E-Cigarette Influence on Instagram With Deep Learning and Text Analysis
Source: Front Commun (Lausanne). Author manuscript; Available in PMC 2022 Feb 28. (PMC8883232; doi:10.3389/fcomm.2019.00075)
Supplement: Supplementary Figure3 [file NIHMS1776832-supplement-Supplementary_Figure3.pdf]

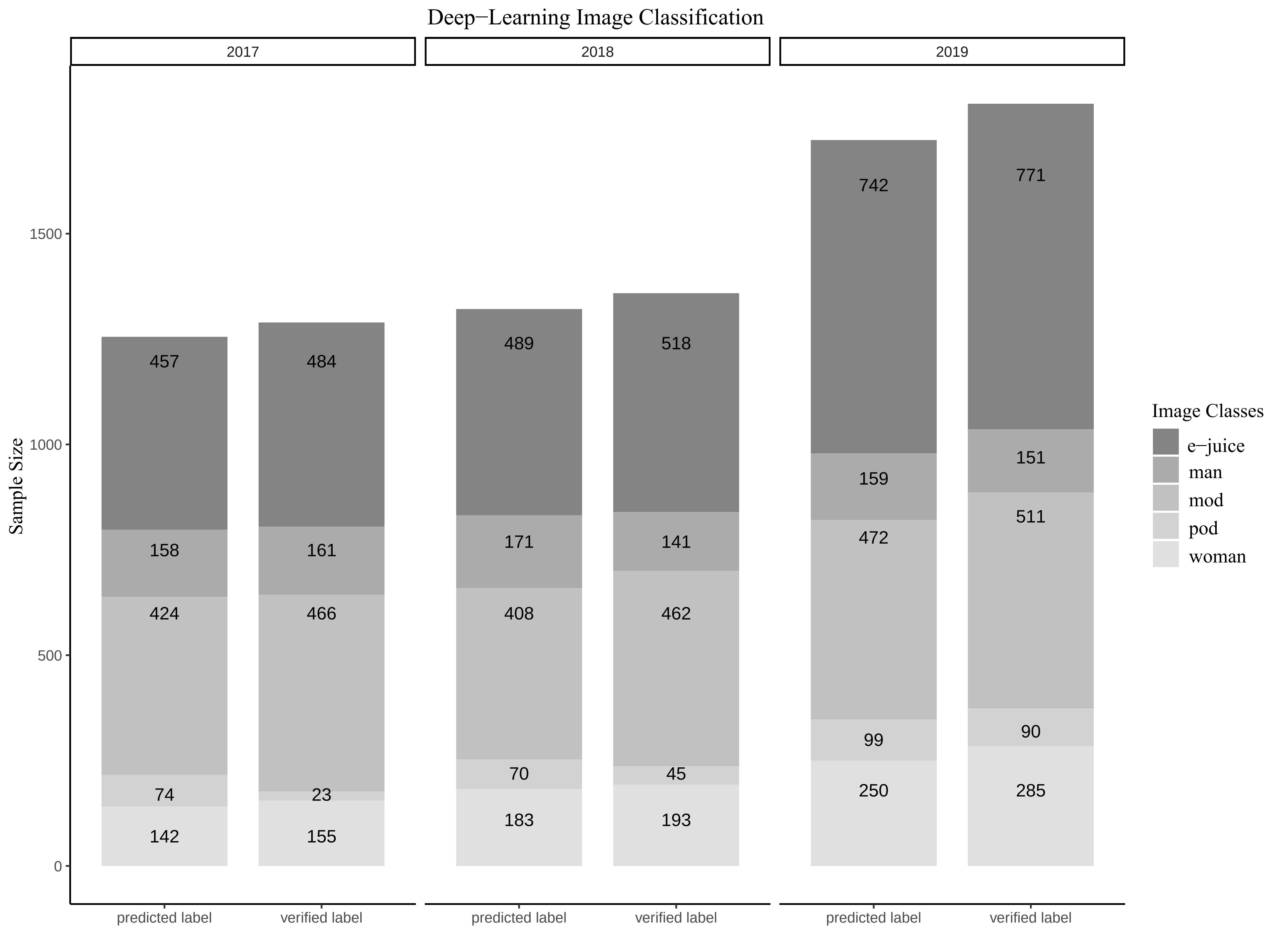

Supplementary Figure 3. Proof of concept image classification was performed on 4,956 posts with the highest like count: likes > 30 in the 2017 and 2018 samples; likes > 100 in the 2019 sample. Class "other" is not included in the figure.
