# Supplementary material for: #Vape: Measuring E-Cigarette Influence on Instagram With Deep Learning and Text Analysis
Source: Front Commun (Lausanne). Author manuscript; Available in PMC 2022 Feb 28. (PMC8883232; doi:10.3389/fcomm.2019.00075)
Supplement: Supplementary Figure4 [file NIHMS1776832-supplement-Supplementary_Figure4.pdf]

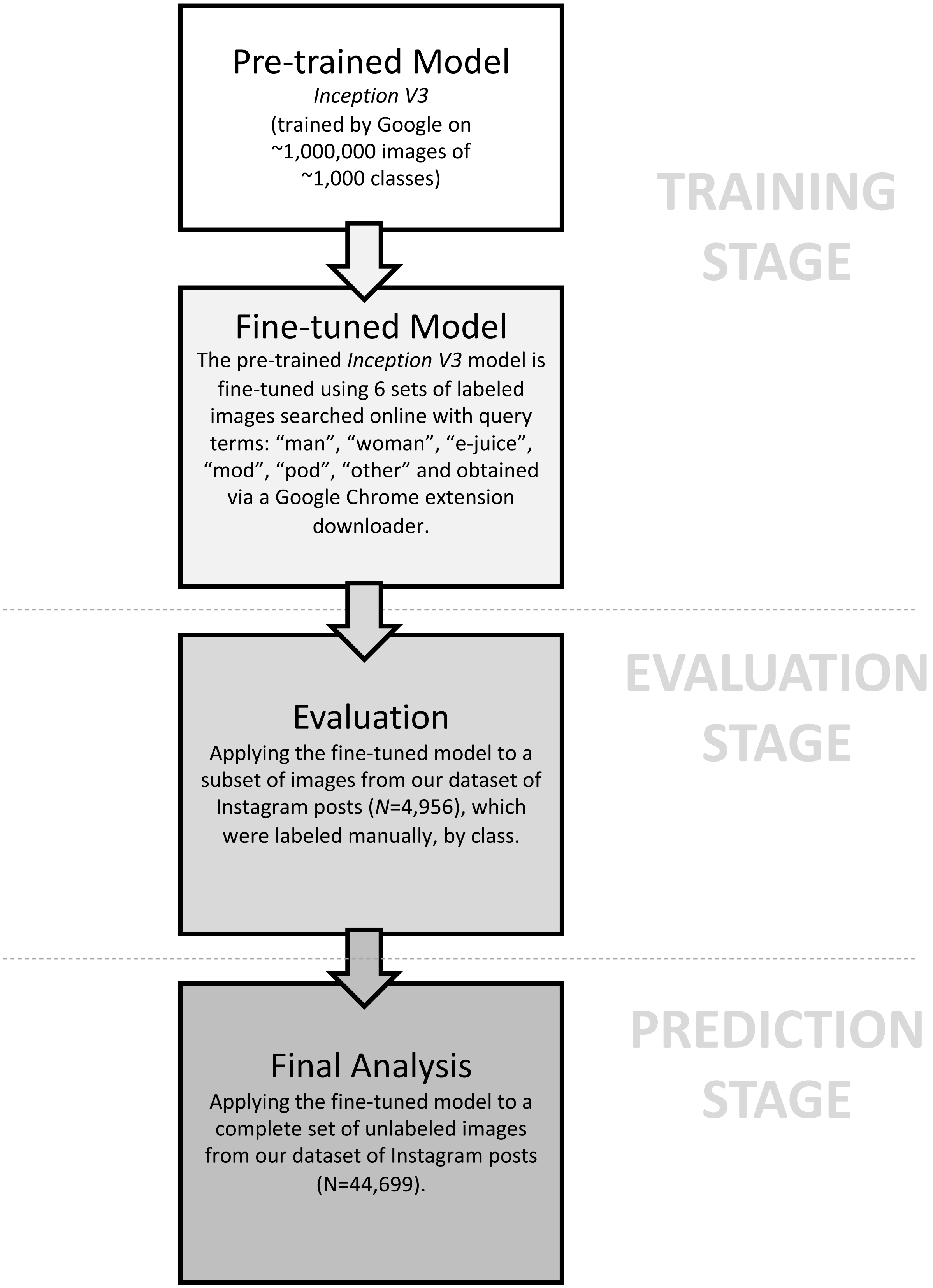

Supplementary Figure 4. Training, evaluation and prediction stages for deep learning image classification.
